# Hearing Difficulties Among Farmworkers in the México-US Southwest Border Region

**DOI:** 10.1007/s10903-024-01592-8

**Published:** 2024-04-22

**Authors:** Laura Coco, Gabriela D. Sanchez, Gabriel A. Campuzano, Annie J. Keeney, James K. Romine

**Affiliations:** 1 School of Speech, Language, and Hearing Sciences, San Diego State University, 5500 Campanile Drive, San Diego, CA 92182 USA; 2 School of Social Work, San Diego State University, 5500 Campanile Drive, San Diego, CA 92182 USA; 3 School of Public Health, San Diego State University, 5500 Campanile Drive, San Diego, CA 92182 USA

**Keywords:** Exposure assessment, Farmer, Hearing loss prevention, Hearing protection, Noise, Occupational health

## Abstract

Migrant and seasonal farmworkers are a vulnerable population with a potentially high risk for hearing loss due to farm-related noise exposures. Occupational noise-induced hearing loss (NIHL) is permanent, and it is associated with an increased risk for injuries on the job, as well as communication difficulties, isolation, and depression. The México/US border region is one of the most productive agricultural regions in the country, however, no known studies have explored hearing loss among farmworkers in this area. This pilot study was a first step toward measuring and addressing hearing loss and noise exposure among this region’s farmworkers. We conducted a cross-sectional survey to estimate the prevalence of subjective hearing difficulties among Yuma County, Arizona farmworkers. Survey interviews took place during a late-night farmworker health fair from 2 am to 6 am to accommodate local farms’ labor schedules. Multivariable regression adjusted for demographic and work covariates estimated subjective hearing loss prevalence ratios. Among 132 farmworker participants, 36% reported they have or might have hearing loss, and 62% reported no hearing loss. Subjective hearing loss prevalence was lower in farmworkers who report not working in noise compared to prevalence in farmworkers who work in noise [prevalence ratio, 0.44 (95% CI 0.23–0.82)]. This report contributes to understanding the perception of hearing-related health and occupational exposures among farmworkers in the México-US Southwest border region. The information from this line of research will inform appropriate safety measures known to lower the risk of experiencing occupational NIHL.

## Introduction

Occupational noise-induced hearing loss (NIHL) is a significant public health problem. It is one of the most common work-related injuries and accounts for 16% of the disabling hearing loss in adults globally [1, 2]. Noise contributes to worker fatigue, stress, and a higher risk for work-related injury [3, 4]. The cumulative effect of noise leads to hearing loss, which is commonly associated with detrimental effects on quality of life, communication, and the daily processes of life, including depression, social isolation, and even an increased risk of developing dementia [5–9].

Migrant and seasonal farmworkers are a vulnerable population that is at a potentially high risk for occupational NIHL [10]. Farming activities that present high noise levels include the operation of machinery, such as power tools, tractors without cabs, and older cabbed tractors, as well as the handling of livestock [11, 12]. For example, past studies have documented that farm tractor noise can reach between 90 and 100 dBA [12]. A worker who is regularly exposed to such levels is at a high risk for NIHL [13]. Data on the exposure levels associated with tasks and equipment on farms in the Mexico-United States Southwest border region are lacking. However, the equipment involved (e.g., tractors, harvesters, conveyor belts) likely produce hazardous noise levels. In addition to intense noise, farmworkers’ hearing may be damaged by chemicals such as herbicides and pesticides known to be ototoxic agents [14]. Co-exposure to both noise and pesticides may result in a greater risk of hearing loss through a synergistic effect [15–17].

To protect workers from hazards, the Occupational Safety and Health Administration (OSHA) provides a hierarchy framework which identifies and ranks possible interventions. The levels of protection, from most to least effective, include engineering and administrative solutions, such as removing noise hazards and minimizing worker exposure time, followed by enforcing the use of hearing protection [18]. However, hearing loss prevention programs are rarely enforced in agriculture [19].

Clinically, hearing loss is typically evaluated using pure-tone audiometric testing, which involves a licensed audiologist or other related professional presenting tones at different intensities and frequencies to a patient who is listening under headphones via a calibrated audiometer [20]. Pure-tone audiometry requires a quiet testing environment with minimal background noise to avoid the risk of elevated thresholds or false positives. Thus, a sound-attenuated room (audiometric booth) is typically used to ensure ambient noise values are within specified ANSI standards [21]. The calibrated equipment, audiometric booth, and trained personnel needed to conduct a hearing evaluation can prohibit its use in community-based settings [22] and poses a challenge to hearing loss research among farmworkers.

Despite the challenge of carrying out pure-tone audiometric testing in non-clinic settings, a few regional studies have been conducted among farmworkers, helping advance our understanding of the NIHL risk in this population. For example, Thelin and colleagues (1983) measured the hearing loss of farmers (n = 161) and non-farmers (n = 75) at the 1979 Missouri Farmers Association Agricultural Fair, and found that farmers had poorer hearing at 2,000 and 4,000 Hz for every 10-year age group from 25 to 64 years [23]. Similarly, Marvel and colleagues (1991) compared hearing screening outcomes between 49 New York dairy farmers and 49 non-farmers and found that farmers had a higher rate of high-frequency hearing loss (65%, n = 32) compared to the non-farmer controls (12%, n = 6) [24]. At a regional farm show in the Midwest, Kerr et al. conducted screening audiograms among farmworkers (n = 150) and compared outcomes with construction workers (n = 147) [19]. Results of that study indicated that more farmworkers had hearing loss than construction workers at 4,000 Hz (67% vs. 53%, respectively).

Self-reported hearing loss is a screening method frequently used in community-based studies in which it is challenging or impossible to carry out pure-tone testing [25] and in population-based epidemiologic studies [26]. In one of the most significant hearing loss studies in agriculture, Gomez and colleagues collected self-report data via telephone interviews among 1,727 farmworkers and farm residents in New York [27]. Results indicated that 36% of respondents endorsed having subjective hearing loss and, among a subset of individuals who completed audiograms (n = 376), nearly half had high-frequency hearing loss. However, one major limitation of this study is that the authors intentionally excluded migrant and seasonal farmworkers. This limitation is important to consider given that the majority of the United States agricultural workforce is comprised of this population [28]. Similarly, few occupational NIHL studies include populations of Latino and Spanish-speaking farmworkers, the demographics of which reflect the vast majority of hired farmworkers in the United States [29].

In one exception, Rabinowitz and colleagues conducted a cross-sectional survey of hearing loss among migrant and seasonal farmworkers in Connecticut (n = 150) [30]. Data were collected over two years (2001–2002) using self-report surveys and pure-tone audiometric testing from a mobile van. Results indicated that 52% (n = 78) of farmworkers had high-frequency hearing loss, and 35% (n = 53) had self-reported difficulty hearing or understanding speech. Further, Hispanic farmworkers had more than three times greater odds of self-reported hearing difficulties than non-Hispanic farmworkers (*p* = 0.02), leading the authors to suspect that language barriers experienced in daily life may exacerbate subjective hearing problems. These results help emphasize the need for additional research among farmworkers from diverse ethnic and language backgrounds, and yet, over the past two decades, little work has been done in this area. In addition, NIHL research among farmworkers in the México-US Southwest border region is lacking despite this being one of the most agriculturally productive regions in the US [31].

A crucial step towards preventing NIHL involves understanding the extent of noise exposure in the target region’s population, mainly because agricultural practices, and thus noise exposures, are different in each area. For example, the primary crop in Connecticut, illustrated in Rabinowitz et al., is tobacco, whereas in the US Southwest, a major crop is lettuce. The equipment and tasks, and thus exposures, involved in these two crops’ pre-harvest, harvest, and production are entirely different. In addition, the majority of previous NIHL studies on farmworkers are outdated. Given changes in agricultural practices over time, including increased mechanization and the demographics of farm labor, more recent studies are warranted [32]. Also, past studies took place prior to the COVID-19 pandemic. Since then, the use of face masks has become more common. Face masks can negatively impact speech recognition abilities [33] potentially affecting hearing protection use. Therefore, updated information is needed.

This pilot study is a preliminary step toward a long-term, cross-disciplinary, community-engaged study of occupational exposures among farmworkers in the México-US Southwest border region. The report aims to describe the results of a survey on subjective noise exposure, hearing difficulties, use of hearing protection, and use of pesticides among farmworkers working in farms in this region.

## Methods

### Setting and Context

This study took place in Yuma, Arizona. Data collection happened over four hours at a health fair for farmworkers. The health fair is carried out annually by a nonprofit organization that provides information and access to health and social services for farmworkers and their families. The event offers multiple health services, including diabetes screenings, cancer screenings, immunizations, and information on resources and donations such as blankets, food, and clothes.

Yuma County is in the southwest corner of Arizona and borders California and the Mexican states of Sonora and Baja California. Given its proximity to the international border, many farmworkers regularly commute to agricultural jobs from their homes in Mexico. Farms in Yuma County plant and harvest diverse crops, including over 175 varieties of leaf vegetables, citrus fruit, and grains [34]. Due to a long growing season made possible by the warm climate, farms in Yuma County produce nearly all of the leafy green vegetables consumed in the United States during winter—including lettuce, cabbage, kale, and spinach [35]. Because of this, Yuma is known colloquially as the “Winter Salad Bowl Capital” of the US, and farmworkers in Yuma are often referred to as *lechugueros*, or people who harvest lettuce [36].

### Survey Development

The items on the survey were adapted from previous questionnaires that have shown to be good predictors of audiometric hearing loss. We included the screening question “¿Cree usted que podría tener pérdida auditiva? (Do you think you might have a hearing loss?)”. This single-item question has shown to be an accurate and sensitive self-report tool with sensitivity ranging from 67 to 76% and specificity ranging from 73 to 92% [37–39]. The 9-item survey was developed simultaneously in Spanish and English by bilingual and bilingual/bicultural researchers and checked for linguistic accuracy by community partners.

### Survey Procedures

Recruitment of volunteers and data collection took place in-person at the Día del Campesino health fair on December 3, 2022. The health fair took place in the early morning (from 2:00 am to 6:00 am) to accommodate farm work schedules. Individuals ages 18 and up who self-reported they were farmworkers were invited to participate in the survey. After being offered a consenting document and providing informed verbal consent, farmworkers completed a brief 9-item survey regarding: subjective hearing difficulties; use of hearing protection; noise in the workplace; as well as use of pesticides. Surveys were available in English and Spanish, although surveys were completed in Spanish. Due to the fast pace of the health fair, all surveys were carried out orally, with researchers asking participants survey questions aloud interview-style. The Institutional Review Board at San Diego State University reviewed and approved the study protocol and survey instrument. No incentive was provided for participating in the survey, although all health fair attendees were offered information on NIHL and hearing protection devices, samples of foam earplugs, and verbal instructions on how to use them.

### Statistical Analysis

The primary sample for this analysis included 132 participants, excluding those with missing data on hearing-loss status (n = 2) and leaving 130 observations for univariate analyses. Missing data for some covariates reduced the sample for multivariable models to a minimum of 125 observations. The covariate with the most missingness was gender (n missing = 4).

To examine univariate (unadjusted) associations of demographic or occupational variables with prevalent subjective hearing difficulties, we displayed proportions (categorical covariates) or medians with interquartile range (continuous covariates) for both categories of subjective hearing loss status (“yes/unsure” or “no”). The distribution of years of agricultural work was right skewed, therefore we displayed medians with the 5th and 25th percentile values.

To further examine associations with subjective hearing difficulties, we arbitrarily divided age into 18–30, 31–45, 46–60, and 61–80 years, as well as years of agricultural work experience into 0.25–10, 11–20, 21–30, 31–40, and 41–60 years. Participants with the fewest years of age or work experience were contrasted as reference categories against those with greater years. For hearing loss-related risk factors, we defined reference categories as those that affirmed exposure (“yes,” “daily,” or “always”) contrasted with those that negated exposure (“never”) or suggested some exposure (“sometimes”).

We modeled prevalent subjective hearing difficulties by fitting log-binomial regression models. From these models, we estimated multivariable adjusted proportion ratios (“prevalence ratios”) of hearing loss and their 95% confidence intervals. Each regression model was fit with three sets of predictors: (1) a single independent variable (unadjusted association); (2) all demographic variables (age, gender, or years of agricultural work) *or* all risk factors for hearing loss (job noise, loud machinery, hearing protection use, and pesticides); and (3) all demographic variables and all risk factors for hearing loss. The analysis software was SAS®.

## Results

**Fig. 1:**
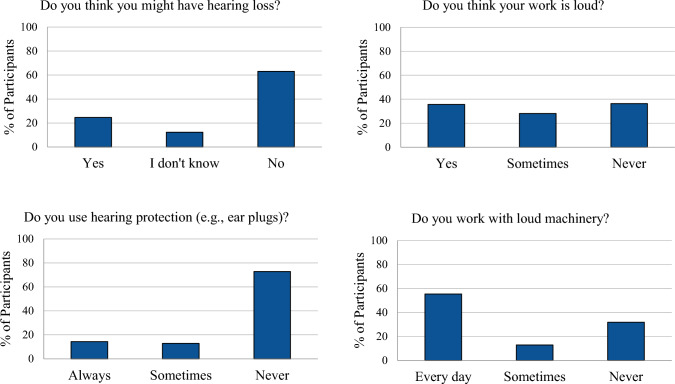
Survey answers regarding hearing loss, hearing protection use, and experience with noise at work among farmworkers in the México-US Southwest border region

Of 132 farmworkers who took part in this study, 45 (36%) reported they have or might have hearing loss, and 82 (62%) reported no hearing loss. The remainder (n = 2) were excluded because they did not provide information on hearing. Figure 1 shows the distribution of survey responses according to NIHL-related questions, and Table 1 shows the demographic and occupational variables distribution across the entire cohort and separated groups. Overall, there were similar numbers of males and females, and most farmworkers reported their ethnicity as Hispanic/Latino. The median age of all farmworker participants was 52 years (range: 18–81 years), and the median length of time in agriculture was 16 years (range: 3 months to 60 years). As seen in Table 1, farmworkers with self-reported hearing loss were more likely to report having a loud job.

**Table 1:** Demographic or occupational variables by self-reported hearing loss status among farmworkers attending a health fair

Variable	All n = 132	Subjective hearing loss^a^	
		Yes/unsure n = 48 (36%)	No n = 82 (62%)
Age, years, percentiles: 25th, 50th, 70th	34, 52, 60	35, 54, 60	33, 51, 58
Gender, %			
Female	61 (46.21)	23 (47.92)	38 (46.34)
Male	67 (50.76)	23 (47.92)	42 (51.22)
Missing	4 (3.03)	2 (4.17)	2 (2.44)
Hispanic/Latino, %			
Yes	117 (88.64)	42 (87.50)	73 (89.02)
No	3 (2.27)	0	3 (3.66)
Missing	12 (9.09)	6 (12.50)	6 (7.32)
Agricultural work, years, percentiles: 5th, 25th, 50th	2, 7, 16	3, 9, 16	2, 7, 17
Job noise, %			
Yes	47 (35.61)	22 (45.83)	24 (29.27)
Sometimes	37 (28.03)	16 (33.33)	20 (24.39)
Never	48 (36.36)	10 (20.83)	38 (46.34)
Loud machines, %			
Daily	73 (55.30)	29 (60.42)	43 (52.44)
Sometimes	17 (12.88)	7 (14.58)	10 (12.20)
Never	42 (31.82)	12 (25.00)	29 (35.37)
Hearing protection, %			
Always	19 (14.39)	5 (10.42)	14 (17.07)
Sometimes	17 (12.88)	8 (16.67)	9 (10.98)
Never	96 (72.73)	35 (72.92)	59 (71.95)
Pesticides, %			
Yes	83 (62.88)	33 (68.75)	49 (59.76)
Sometimes	12 (9.09)	5 (10.42)	7 (8.54)
Never	36 (27.27)	9 (18.75)	26 (31.71)
Missing	1 (0.76)	1 (2.08)	(0.00)

Percentages may not add up to 100 because of rounding

^a^Measured using the single item: "Do you think you might have a hearing loss?" [37–39]; n = 2 missing

Tables 2 and 3 show unadjusted and adjusted prevalence ratios of subjective hearing loss (yes/unsure compared to no) relative to referent categories. As seen in Table 2, the prevalence of self-reported hearing loss was not monotonically associated with years of agricultural work experience: 0.25–10 years: 33%; 11–20 years: 43%; 21–30 years: 44%; 31–40 years: 25%; 41–60 years: 42%. The relation of age with subjective hearing loss also did not show a consistent pattern. Similarly, the prevalence of self-reported hearing loss in females was only 3 percentage points higher compared to men (38% vs. 35%).

**Table 2:** Prevalence and prevalence ratios of subjective hearing loss by age, gender, or years of agricultural work

	Age, years				Sex		Agricultural work, years				
	18–30	31–45	46–60	61–81	Female	Male	0.25–10	11–20	21–30	31–40	41–60
	(n = 20)	(n = 32)	(n = 49)	(n = 28)	(n = 61)	(n = 67)	(n = 49)	(n = 30)	(n = 18)	(n = 20)	(n = 12)
Prevalence	35.0%	28.1%	40.8%	39.3%	37.7%	35.4%	32.7%	43.4%	44.4%	25.0%	41.7%
Prevalence ratio	Ref	0.80	1.17	1.12	Ref	0.94	Ref	1.33	1.36	0.77	1.28
95% confidence limits		(0.36–1.81)	(0.59–2.32)	(0.53–2.39)		(0.59–1.49)		(0.75–2.36)	(0.71–2.62)	(0.32–1.81)	(0.58–2.79)
Model 1^a^											
Adjusted prevalence ratio	Ref	0.71	1.43	1.12	Ref	0.95	Ref	1.20	1.11	1.00	1.40
95% confidence limits		(0.31–1.64)	(0.74–2.78)	(0.50–2.47)		(0.60–1.51)		(0.68–2.15)	(0.56–2.21)	(0.43–2.34)	(0.59–3.32)
Model 2^b^											
Adjusted prevalence ratio	Ref	0.66	1.48	1.40	Ref	0.82	Ref	0.96	0.85	0.88	1.16
95% confidence limits		(0.27–1.58)	(0.75–2.93)	(0.56–3.51)		(0.46–1.45)		(0.49–1.88)	(0.37–1.96)	(0.35–2.20)	(0.42–3.27)

^a^Covariates that are presumed risk factors for hearing loss: job noise, loud machines, hearing protection use, pesticides

^b^Covariates from model 1 and covariates that are demographic variables: age, gender, years of agricultural work

**Table 3:** Prevalence and prevalence ratios of subjective hearing loss by occupational hearing-related variables

	Job is loud			Loud machines			Hearing protection			Pesticides		
	Yes	Sometimes	Never	Daily	Sometimes	Never	Always	Sometimes	Never	Yes	Sometimes	Never
	(n = 46)	(n = 36)	(n = 48)	(n = 72)	(n = 17)	(n = 41)	(n = 19)	(n = 17)	(n = 94)	(n = 82)	(n = 12)	(n = 35)
Prevalence	47.8%	44.4%	20.8%	40.3%	41.2%	29.3%	26.3%	47.1%	37.2%	40.2%	41.7%	25.7%
Prevalence ratio	Ref	0.93	0.44	Ref	1.02	0.73	Ref	1.79	1.41	Ref	1.04	0.64
95% confidence limits		(0.58–1.49)	(0.23–0.82)		(0.54–1.93)	(0.42–1.26)		(0.72–4.42)	(0.64–3.14)		(0.50–2.13)	(0.34–1.19)
Model 1^a^												
Adjusted prevalence ratio	Ref	0.97	0.43	Ref	0.94	0.66	Ref	1.84	1.66	Ref	1.01	0.65
95% confidence limits		(0.60–1.59)	(0.23–0.81)		(0.47–1.90)	(0.37–1.16)		(0.66–5.12)	(0.68–4.09)		(0.49–2.07)	(0.34–1.24)
Model 2^b^												
Adjusted prevalence ratio	Ref	0.91	0.35	Ref	0.85	0.76	Ref	1.90	1.66	Ref	0.83	0.71
95% confidence limits		(0.52–1.59)	(0.15–0.82)		(0.37–1.99)	(0.42–1.37)		(0.58–6.19)	(0.63–4.41)		(0.40–1.71)	(0.37–1.37)

^a^Covariates that are demographic variables: age, gender, or years of agricultural work

^b^Covariates from model 1 and covariates that are presumed risk factors for hearing loss: job noise, loud machines, hearing protection use, or pesticides

As shown in Table 3, as expected, those who reported never experiencing select risk factors for hearing loss were also less likely to have subjective hearing loss. The prevalence ratio and 95% confidence intervals of those reporting unexposed (versus exposed) to excess job noise were 0.44 (0.23–0.82). Multivariable adjustment did not identify any other statistically significant relationships, although there were several associations worth noting. The prevalence ratio and 95% confidence intervals of farmworkers who work with loud machines or pesticides were 0.73 (0.42–1.26) and 0.64 (0.34–1.19), respectively. Similarly, the prevalence of reporting hearing loss (yes/unsure compared to no) was 11 percentage points lower among those who reported always (compared to never) using hearing protection (26% vs. 37%).

## Discussion

Farmworkers are at a high risk for work-related injury, illness, and death due to prolonged heat, dust, chemical use, and machinery operations [40]. Many farmworkers also experience a disproportionately high rate of social hazards, such as food insecurity, lack of quality housing, and fear of deportation [41]. Occupational NIHL is a commonly overlooked risk even though it can increase the likelihood of work-related injuries [4]. Numerous other negative consequences are widely associated with hearing loss, including an increased risk for social isolation, depression, negative quality of life, and cognitive decline [5, 6, 42]. Data on farmworkers’ hearing loss is sparse because this is a particularly vulnerable, hard-to-reach, and mobile population. This pilot study represents an initial step in obtaining information on the hearing abilities of primarily Latino farmworkers in the México/US Southwest border region, a population that has not yet been represented in the NIHL literature, despite this being one of the most productive agricultural regions in the US.

The findings from this pilot study indicate that 36% of farmworkers in our sample have or might have subjective hearing loss. These results are similar to past research that has described rates of self-reported hearing difficulties among populations of farmworkers in New York (36%), Kentucky (36%), Missouri (47%), New England (39%), and the Midwest (39%) [19, 23, 27, 30, 43]. Additional past research using audiometric testing has observed hearing loss in between 28 and 50% of farmworkers [27, 44, 45] and 28–57% of farm operators (managers), depending on the frequency tested. As mentioned, many existing studies on farmworkers are over 20 years old. Given changes in agricultural practices over time, including increased mechanization, more recent studies are warranted.

The reported use of hearing protection in this study (27%) is higher than in some previous reports [30, 43, 46]. For example, Carruth and colleagues found that, among 56 farmworkers and family members surveyed in Louisiana, only four people (7%) reported wearing hearing protection more than 50% of the time. In Gates and Jones, 12% (3 of 25) of farmworkers in Kentucky reported either always, often, or sometimes using hearing protection [43]. A slightly higher prevalence (33%) of reported use of hearing protection was observed in a 1992 telephone survey of a large cohort (n = 1,947) of California farmworkers [47]. There is conflicting evidence in the literature regarding the reliability of self-reported hearing protection data. One study found that farmworkers’ reported use of hearing protection is higher than observed [48]. However, another study concluded that self-reporting is a valid way to measure hearing protection use because reporting hearing protection use does not appear to be impacted by social desirability bias [49]. In addition, researchers have identified a correlation between low acculturation and high perceived barriers to hearing protection among Latino industrial workers [50]. The current study did not collect data on barriers to hearing protection use, although this will be explored in future steps. Given that this study found higher proportion of self-reported hearing protection use than what has been reported in the literature, future research will also explore the facilitators to hearing protection use in this population.

The farmworkers in this study had a wide range of ages, although many (31%) were younger than 40 years of age. If NIHL starts in adolescence, it could result in disability over a lifetime [10, 51]. Agriculture is a unique industry in which children as young as 10 years old are allowed to be employed [52]. Thus, efforts to prevent NIHL among farmworkers are particularly important. NIHL is permanent and irreversible; thus, developing strategies for better prevention, early detection, and intervention is crucial.

## Strengths, Limitations, and Future Needs

A significant strength of this study is its focus on an underrepresented, vulnerable population of Spanish-speaking, primarily Hispanic/Latino farmworkers on the México/US border. Hearing conservation research among farmworkers is limited, perhaps partly because this is considered a “hard to reach” population [10, 53, 54]. This may be particularly true among Mexican migrant and seasonal farmworkers in the México/US Southwest border region who often move across international boundaries daily [55]. Many agricultural NIHL studies either do not include Hispanic/Latino farmworkers [19], do not report participants’ race/ethnicity and language [43, 56], or intentionally exclude farmworkers who do not speak/read in English [57, 58]. This limitation severely impacts the extent to which findings are generalizable and scalable. Such representative research is fundamental given that past research has observed barriers to hearing protection use based on acculturation [50, 59]. In addition, there are no known studies of farmworkers’ hearing in the US Southwest region. As mentioned, regional studies are highly valuable because they reflect the area’s exposures, helping contribute to appropriate hearing conservation programs and allowing for comparison with other studies.

Another strength of this study is our collaboration with a community organization, which has been recommended in agricultural research [43]. This survey study was made possible due to a partnership with a community-driven farmworker organization. Notably, OSHA, a federal agency, has specifically excluded most farmworkers from their noise standards since its formation in 1971 [60], and many state programs responsible for occupational safety and health also lack agricultural noise standards. Without federal and state regulations, information on the prevention of NIHL must come from other sources, including community organizations, farm managers, extension offices, and other agencies. As per the OSHA hierarchy of controls framework, employers should prioritize engineering controls, such as replacing current noisy equipment with quieter alternatives. At a minimum, protective hearing equipment (e.g., ear plugs, earmuffs) should be provided at worksites, and workers should be instructed on proper use and encouraged to wear them when in noise.

The limitations of this study include the convenience sampling approach; the participants who volunteered for this study may have had an interest in or concern about their hearing. Also, this study focused on self-report survey data, and thus, the data are susceptible to bias from inaccurate recall and/or social desirability. While some researchers have found that a brief survey of farmworkers’ self-perceived hearing loss can be a good predictor of audiometric hearing loss [27], others have found the opposite [19]. Thus, follow-up studies will involve collecting objective information, including audiometric hearing assessments and noise dosimetry, to describe further farmworkers’ hearing loss and noise levels on the farm. Given recent emphasis on a “total worker health” approach, future research will also explore hearing loss and noise impacts as they relate to stress, anxiety, and depression in this population.

The survey was intentionally designed to be brief to capture as much quality data as possible among a mobile, time-limited group of workers. Given its brevity, the survey could not capture information such as the individual’s work role, jobs unrelated to farming, recreation activities that involve noise, and the frequency of noise exposure and hearing protection use. Hearing loss frequently arises as a natural consequence of the aging process, and therefore, subjective reports of hearing loss in this sample may not be a result from noise exposure alone. Another limitation to consider is that, due to the data collection procedure, a response rate could not be collected. However, the research team noted that farmworkers who declined participation were rushed for time. Future studies may capture more detailed information by taking place in other locations, such as farmworkers’ homes, work transit sites and workplaces, to allow time for additional data collection measures.

## Conclusion

To the best of our knowledge, this is the first study of NIHL among farmworkers who work on farms in the México/US Southwest border region. It adds to the handful of studies on noise exposure in agriculture in other areas. Given the high proportion of farmworkers in this study who reported working in noise and the low number of people who reported use of hearing protection, additional research is warranted. Future work will involve objective measures of hearing loss and noise exposure, as well as longer-term goals that include developing culturally situated hearing conservation practices to help reduce risk of NIHL. Among the many health hazards precarious employees are at risk for, noise exposure may be one of the most actionable for occupational health and safety efforts.
